# *Fusarium nirenbergiae* (*Fusarium oxysporum* Species Complex) Causing the Wilting of Passion Fruit in Italy

**DOI:** 10.3390/plants10102011

**Published:** 2021-09-26

**Authors:** Dalia Aiello, Alberto Fiorenza, Giuseppa Rosaria Leonardi, Alessandro Vitale, Giancarlo Polizzi

**Affiliations:** Dipartimento di Agricoltura, Alimentazione e Ambiente, sez. Patologia Vegetale, University of Catania, Via S. Sofia 100, 95123 Catania, Italy; dalia.aiello@unict.it (D.A.); alberto.fiorenza.93@gmail.com (A.F.); leonardigiusi@outlook.it (G.R.L.); gpolizzi@unict.it (G.P.)

**Keywords:** wilt, passion fruit, *Fusarium oxysporum* species complex

## Abstract

Passion fruit (*Passiflora edulis* Sims.) is an ever-increasing interest crop in Italy because it is mainly cultivated for its edible fruit and, secondly, as an ornamental evergreen climber. During the summer of 2020, two-year-old plants of purple passion fruit in one of the most important expanding production areas of Sicily (southern Italy) showed symptoms of yellowing, wilting, and vascular discoloration. *Fusarium*-like fungal colonies were consistently yielded from symptomatic crown and stem tissues. Five representative isolates were characterized by a morphological and molecular analysis based on a multilocus phylogeny using RNA polymerase’s second largest subunit (RPB2) and translation elongation factor 1-alpha (EF-1α) genes, as *Fusarium nirenbergiae* (*Fusarium oxysporum* species complex). Pathogenicity tests conducted on healthy 1-year-old passion fruit cuttings revealed symptoms similar to those observed in the field. To our knowledge, this is the first report of Fusarium wilt on passion fruit caused by *Fusarium nirenbergiae.* This report focuses on the phytopathological implications of this fungal pathogen, which may represent a future significant threat for the expanding passion fruit production in Italy and Europe.

## 1. Introduction

In recent years, tropical fruit production increased worldwide due to the increasing demand of global markets and more efficient transportation and storage techniques [1,2]. Most of the tropical fruit is destined for fresh consumption or industrial transformation. Among these, passion fruit (*Passiflora edulis* Sims.) is one of the most exported and consumed fruit commodities. It originated in tropical and subtropical America [3], and it is now extensively cultivated worldwide, including Australia, New Zealand, India, Africa, and South America [4,5]. Passion fruit is mainly cultivated for its edible fruit but secondarily also for its attractive flowers on ornamental evergreen vines.

In Italy, the cultivation of *P. edulis* (also known as purple passion fruit) as a fruit crop in some regions characterized by a Mediterranean climate (e.g., Sicily and Calabria) is gaining growing interest by local farmers, and it is carried out under greenhouse and, to a lower extent, open field conditions. Indeed, although the crop is well adapted to a wide rainfall range (1000–2500 mm for crop season), minimum temperatures below 5 °C should be avoided because they seriously compromise the plant growth and nutrient uptake [6,7,8]. In this regard, it should be noted that a process of reconversion of protected tomato and vegetable crops into tropical fruit plantations is currently taking place in southern Italy and Sicily.

Unfortunately, this species is affected by many diseases during its different growth stages, and this reduces the yield and the farmers’ income [9]. One of the most widely reported fungal pathogens affecting passion fruit is *Fusarium oxysporum* f. sp. *passiflorae*, which causes the Fusarium wilt. It was first reported in Australia [10] but is nowadays spread worldwide [11,12,13]. Among Fusarium diseases, *Neocosmospora solani* (=*Fusarium solani*) is responsible for the basal stem rot [14,15,16]. According to Viana & Costa [17], the species *F. oxysporum* f. sp. *passiflorae* and *N. solani* are the most damaging ones to passion fruit crops. Minor diseases have been reported on passion fruit, such as the damping-off of seedlings and collar and root rot in adult plants caused by *Rhizoctonia solani* [18] and collar rot caused by *Phytophthora* spp. [19]. During a recent survey performed in Sicily, young passion fruit plants showing symptoms of general yellowing and wilting were observed in some of the most representative production areas. Given the increasing interest of local growers in expanding passion fruit cultivation, the aim of this study was to characterize the fungal species associated with those symptoms and test their pathogenicity, in order to better understand the syndrome’s aetiology.

## 2. Results

**Figure 1:**
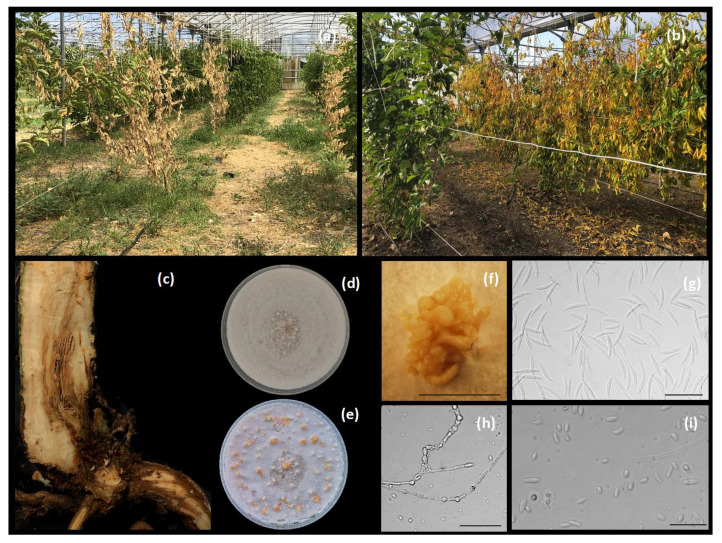
Disease symptoms and *Fusarium nirenbergiae* features: (**a**,**b**), yellowing and wilting of passion fruit plants in greenhouse; (**c**), vascular discoloration on a collar portion; (**d**,**e**), *F. nirenbergiae* (Di3A-Pef1 isolate) grown on 7 day-old (up) and 14 day-old (down) OA; (**f**), sporodochia on OA; (**g**), sporodochial conidia (macroconidia); (**h**), chlamydospores on SNA; (**i**), aerial conidia (microconidia). Scale bars, (**f**): 2 mm; (**g**–**i**): 50 μm.

The symptoms observed in the greenhouse consisted of leaf yellowing and wilting (Figure 1a,b), external crown and root rot and wood discoloration moving upward to the canopy (Figure 1c). The disease incidence reached 10% of the cultivated plants. Colonies with white or light purple aerial mycelia and violet pigmentation on the underside of the cultures developed after 14 days on PDA, being firstly identified as *Fusarium*-like. Sporodochial macroconidia with 2 to 5 septa, grown on OA, measured (23.09–) 28.76 ± 3.06 (–35.48) μm × (1.99–) 3.84 ± 0.58 (–4.75) μm (Figure 1f,g). Oval, unicellular microconidia developed on short monophialides, grown on OA, measured (3.1–) 5.17 ± 1.35 (–9.17) μm × (1.3–) 1.98 ± 0.37 (–2.9) μm (Figure 1i).

PCR edit amplicons resulted in 528 bp for the partial ITS region, 287 bp for EF-1α and 953 bp for RPB2. The sequences were registered in GenBank as follows: MZ398141, MZ398142, MZ398143, MZ398144, MZ398145 for ITS, MZ408109, MZ408110, MZ408111, MZ408112, MZ408113 for RPB2 and MZ408114, MZ408115, MZ408116, MZ408117, MZ408118 for EF1-α. A GenBank BLASTn analysis and a pairwise sequence alignment on the MLST database indicated that all the isolates from passion fruit belonged to the *Fusarium oxysporum* species complex (FOSC). In particular, the MLST search resulted in high identity values (96–100%) (Acc. number MH582354) for the EF1-α gene and 98% (Acc. number MH582140) for the RBP2 gene with a *F. oxysporum* species complex (FOSC). The MP heuristic search resulted in 83 parsimony-informative characters, while 109 were variable and parsimony-uninformative and 1412 were constant. A maximum of 320 equally most parsimonious trees were retained (Tree length = 249, CI = 0.851, RI = 0.898 and RC = 0.765).

**Figure 2:**
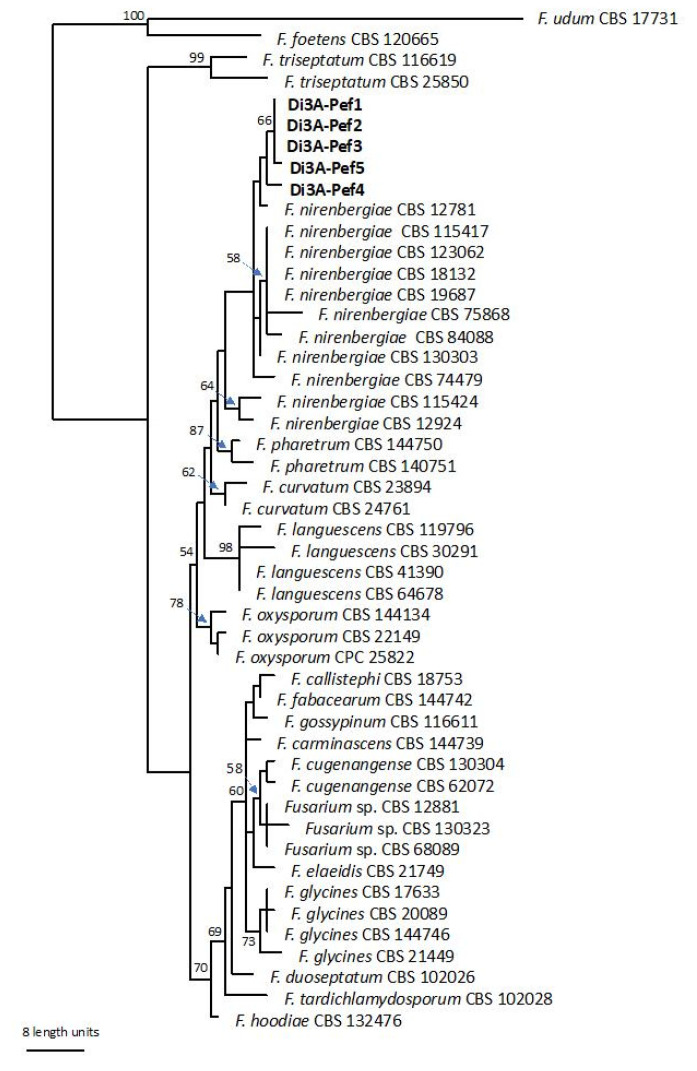
Single most parsimonious phylogenetic tree resulting from the MP analysis of combined *EF1-α* and *rbp2* sequence data. The isolates in bold were sequenced in this study. The numbers represent MP bootstrap values.

The bootstrap support values from the parsimony analysis are shown close to the branch node. The group of representative isolates Di3A-Pef1-5 clustered with the reference strain of *F. nirenbergiae*, as shown in Figure 2, and were clearly separated by the other sequences provided in the study by Lombard et al. [20]. The isolates were then identified as *Fusarium nirenbergiae* L. Lombard & Crous.

The inoculated isolate after five months caused symptoms similar to those observed under greenhouse conditions in all inoculated plants. The symptoms consisted of leaf yellowing and wilting. After 7 months all plants died. A longitudinal section of the inoculated plants reveals the internal discolorations moving upward to the canopy. The control remains symptomless. From the symptomatic tissues, *F. nirembergiae* was always re-isolated, and it was characterized as previously described.

## 3. Discussion and Conclusions

To the best of our knowledge, this paper represents the first report of *F. nirembergiae*, belonging to the FOSC complex, as a causal agent of Fusarium wilt of passion fruit. In this regard, both the morphological characterization and the analysis of the ITS, EF1-α and RBP2 sequences allowed us to correctly allocate a representative number of detected strains within the *F. nirenbergiae* group, being distinctly separated by the other taxa, as recently shown by Lombard et al. [20] and Crous et al. [21]. Based on the present findings, *F. nirenbergiae* was strongly grouped in a separated subclade of FOSC, phylogenetically close to *F. curvatum*. Although little information regarding *F. nirembergiae*’s pathogenicity and host range is currently available, except for the study by Zhao et al. [22] on *Acer negundo*, this species (belonging to FOSC) is able to colonize and infect host vascular tissues; for this reason, it is reported worldwide as responsible for Fusarium wilt [14]. The first symptoms consist of leaf yellowing and wilt, followed by the plant’s collapse. This disease is observed in adult and young plants under favorable conditions for the infection development, such as high temperature and humidity and a high potential inoculum in the soil [5,23]. Once this fungal pathogen is established in the field, its control is very difficult, since fungicide application does not result in a significant reduction of the disease amount, and the pathogen can persist in the soil for many years in the absence of the host [14]. Hence, the incidence data are very worrying as regards the nature of the fungal pathogen and dissemination ability of *F. nirenbergiae* under greenhouse conditions. If, on the one hand, protected systems could facilitate the cultivation of purple passion fruit, on the other hand they could aggravate the consequences of this phytopathological issue. Indeed, this could represent a future threat for the expansion of this tropical crop, which is replacing protected tomato cultivation in different areas of southern Italy. Therefore, disease management should be focused mainly on preventative and pathogen exclusion measures, avoiding plantation in areas with a severe history of Fusarium wilt infections or selecting healthy propagation material in combination with adequate agronomic practices. Additionally, other sustainable strategies should include the use of resistant cultivars, as recommended by several authors [24,25]. Comprehensively, the increasing trend of tropical plantations in Italy leads us to focus more on fungal diseases that could represent limiting factors for future production. According to presented data combined with recent findings [20,21], it cannot be excluded that some past reports of *F. oxysporum* f. sp. *passiflorae* could confirm that *F. nirembergiae* is a causal agent of Fusarium wilt. However, further surveys should be performed on *P. edulis* orchards in Italy and worldwide to confirm the new aetiology of the Fusarium wilt of passion fruit and its real diffusion.

## 4. Materials and Methods

### 4.1. Field Survey, Isolations and Morphological Characterization

In July of 2020, 50 two-year-old ‘purple’ passion fruit plants cultivated in a greenhouse in the Syracuse province (Sicily, Italy) appeared stunted, defoliated and severely wilted. Diseased vascular tissues (0.5 cm^2^) were surface-disinfected for 1 min in a 1.2% sodium hypochlorite (NaOCl) solution, rinsed in sterile water, placed on a potato dextrose agar (PDA, Lickson, Vicari, Italy) amended with 0.1 g/L of streptomycin sulphate (Sigma-Aldrich, St. Louis, MO, USA), to prevent bacterial growth, and then incubated at 25 ± 1 °C until fungal colonies were observed. Single-spore isolates were obtained from pure cultures grown on APDA. To induce sporulation, five representative single-spore isolates (named Di3A-Pef1, Di3A-Pef2, Di3A-Pef3, Di3A-Pef4 and Di3A-Pef5) were selected and transferred on a synthetic nutrient-poor agar (SNA) [26], Oatmeal Agar (OA, Difco, Detroit, MI, USA) and PDA for morphological characterization. A total of 50 macro- and micro-conidia were measured (length and width size) using a fluorescence microscope (Olympus-BX61) coupled to an Olympus DP70 digital camera; images and measurements were captured using the software analySIS Image Processing. Conidia sizes are reported as the minimum and maximum in parentheses, and the average is reported with the standard deviation.

### 4.2. Molecular Characterization and Phylogeny

Genomic DNA of the selected isolates (Di3A-Pef 1-2-3-4-5) was extracted using the Gentra Puregene Yeast/Bact kit (Qiagen, Hilden, Germany) according to the manufacturer’s protocol. The internal transcribed spacer of the ribosomal DNA (rDNA-ITS), partial translation elongation factor alpha gene (EF-1α) and RNA polymerase II gene (RPB2) were targeted for PCR amplification and sequencing. The primers used for these regions were: ITS5 and ITS4 for ITS [27], EF1-728F and EF1-986R for EF-1α [28] and 5f2 and 7cr for RPB2 [29]. The PCR products were purified and sequenced in both directions by Macrogen Inc. (Seoul, Korea). The sequences were edited using MEGAX: Molecular Evolutionary Genetics Analysis across computing platforms [30], manual adjustments of alignments were made when necessary and submitted to GenBank. Moreover, the sequences were blasted in the NCBIs GenBank nucleotide database and on the Fusarium MLST database of the Westerdijk Fungal Biodiversity Institute (http://www.westerdijkinstitute.nl/fusarium/, accessed on 21 May 2021). For comparison, 44 additional sequences were selected according to the recent literature [20] (Table 1). The phylogenetic analysis was based on the Maximum Parsimony (MP). The MP analysis was done using PAUP v. 4.0a165 [31]. Phylogenetic relationships were estimated by heuristic searches with 100 random addition sequences. A tree bisection-reconnection was used, with the branch swapping option set to ‘best trees’ only, with all characters weighted equally and alignment gaps treated as the fifth state. The tree length (TL), consistency index (CI), retention index (RI) and rescaled consistency index (RC) were calculated for parsimony and the bootstrap analyses were based on 1000 replicates [32]. *Fusarium foetens* (CBS 120665) and *F. udum* (CBS 12881) served as outgroups.

### 4.3. Pathogenicity Tests

In order to fulfil Koch’s postulates, pathogenicity tests were conducted on one-year-old potted cuttings using the mycelial plug technique. In detail, 18 healthy cuttings were inoculated, removing a piece of bark of the crown root with a scalpel blade and applying a mycelial plug (0.3 cm^2^), taken from a 14-day-old Di3A-Pef 1 isolate, upside down on the wound and subsequently covered with soil to prevent desiccation. The controls consisted of sterile PDA plugs applied as described above to the same number of healthy young plants. Re-isolation attempts were performed from representative inoculated plants.

## Figures and Tables

**Table 1:** Characteristics of *Fusarium* isolates included in the phylogenetic analysis.

Species	Culture Accession	Host/Substrates	Special form	Origin	GeneBank Accession	
					rpb2	EF1-α
*Fusarium callistephi*	CBS 187.53	*Callistephus chinensis*	*callistephi*	The Netherlands	MH484875	MH484966
*F. carminascens*	CBS 144739	*Zea mays*		South Africa	MH484934	MH485025
*F. cugenengense*	CBS 620.72	*Crocus* sp.	*gladioli*	Germany	MH484879	MH484970
*F. cugenengense*	CBS 130304	*Gossypium barbadense*	*vasinfectum*	China	MH484921	MH485012
*F.curvatum*	CBS 247.61	*Matthiola incana*	*matthiolae*	Germany	MH484876	MH484967
*F.curvatum*	CBS 238.94	*Beaucarnia* sp.	*meniscoideum*	The Netherlands	MH484893	MH484984
*F. duoseptatum*	CBS 102026	*Musa sapientum*	*cubense*	Malaysia	MH484896	MH484987
*F. elaeidis*	CBS 217.49	*Elaeis* sp.	*elaeidis*	Zaire	MH484870	MH484961
*F. fabacearum*	CBS 144742	*Zea mays*		South Africa	MH484938	MH485029
*F. foetens*	CBS 120665	*Nicotiana tabacum*		Iran	MH484918	MH485009
*F. glycines*	CBS 144746	*Glycine max*		South Africa	MH484942	MH485033
*F. glycines*	CBS 20089	*Ocimum basilicum*	*basilici*	Italy	MH484888	MH484979
*F. glycines*	CBS 17633	*Linum usitatissium*	*lini*	Unknown	MH484868	MH484959
*F. glycines*	CBS 21449	Unknown		Argentina	MH484869	MH484960
*F. gossypinum*	CBS 116611	*Gossypium hirsutum*	*vasinfectum*	Ivory Coast	MH484907	MH484998
*F. hoodiae*	CBS 132474	*Hoodia gordonii*	*hoodiae*	South Africa	MH484929	MH485020
*F. languescens*	CBS 41390	*Solanum lycopersicum*	*lycopersici*	Israel	MH484890	MH484981
*F. languescens*	CBS 119796	*Zea mays*		South Africa	MH484917	MH485008
*F. languescens*	CBS 30,291	*Solanum lycopersicum*	*lycopersici*	The Netherlands	MH484892	MH484983
*F. languescens*	CBS 646.78	*Solanum lycopersicum*	*lycopersici*	Morocco	MH484881	MH484972
*F. nirembergiae*	CBS 744.79	*Passiflora edulis*	*passiflorae*	Brazil	MH484882	MH484973
*F. nirembergiae*	CBS 115424	*Agothosma betulina*		South Africa	MH484906	MH484997
*F. nirembergiae*	CBS 12924	*Secale cereale*		Unknown	MH484864	MH484955
*F. nirembergiae*	CBS 12781	*Chrysanthemum* sp.	*chrysanthemi*	USA	MH484883	MH484974
*F. nirembergiae*	CBS 130303	*Solanum lycopersicum*	*radicis-lycopersici*	USA	MH484923	MH485014
*F. nirembergiae*	CBS 115417	*Agothosma betulina*		South Africa	MH484903	MH484994
*F. nirembergiae*	CBS 19687	*Bouvardia longiflora*	*bouvardiae*	Italy	MH484886	MH484977
*F. nirembergiae*	CBS 123062	Tulip roots		USA	MH484919	MH485010
*F. nirembergiae*	CBS 18132	*Solanum tuberosum*		USA	MH484867	MH484958
*F. nirembergiae*	CBS 75868	*Solanum lycopersicum*	*lycopersici*	The Netherlands	MH484877	MH484968
*F. nirembergiae*	CBS 840.88	*Dianthus caryophyllus*	*dianthi*	The Netherlands	MH484887	MH484978
*F. oxysporum*	CBS 221.49	*Camellia sinensis*	*medicaginis*	South East Asia	MH484872	MH484963
*F. oxysporum*	CPC 25822	*Protea* sp.		South Africa	MH484943	MH485034
*F. oxysporum*	CBS 144134	*Solanum tuberosum*		Germany	MH484953	MH485044
*F. pharetrum*	CBS 144751	*Aliodendron dichotomum*		South Africa	MH484952	MH485043
*F. pharetrum*	CBS 144750	*Aliodendron dichotomum*		South Africa	MH484951	MH485042
*F. trachichlamydosporum*	CBS 102028	*Musa sapientum*	*cubense*	Malaysia	MH484897	MH484988
*F. triseptatum*	CBS 258.50	*Ipomea batatas*	*batatas*	USA	MH484873	MH484964
*F. triseptatum*	CBS 116619	*Gossypium hirsutum*	*vasinfectum*	Ivory Coast	MH484910	MH485001
*F. udum*	CBS 177.31	*Digitaria ariantha*		South Africa	MH484866	MH484957
*Fusarium* sp.	CBS 12881	*Chrysanthemum* sp.	*chrysanthemi*	USA	MH484884	MH484975
*Fusarium* sp.	CBS 130323	Human nail		Australia	MH484927	MH485018
*Fusarium* sp.	CBS 68089	*Cucumis sativus*	*cucurbitacearum*	The Netherlands	MH484889	MH484980

## Data Availability

The data presented in this study are available on request from the corresponding author.

## References

[1] Ding P., Thomas B., Murphy D.J., Murray B.G. (2017). Tropical fruits. Encyclopedia of Applied Plant Sciences.

[2] Underhill S., Caballero B. (2003). Fruits of Tropical Climates: Commercial and Dietary Importance. Encyclopedia of Food Sciences and Nutrition.

[3] Cervi A.C. (2006). O Gênero Passiflora (Passifloraceae) No Brasil, Es- Pécies Descritas Após o Ano de 1950.

[4] Manicom B., Ruggiero C., Ploetz R.C., de Goes A., Ploetz R.C. (2003). Diseases of passion fruit. Diseases of Tropical Fruit Crops.

[5] Vanderplank J. (1996). Passion Flowers.

[6] Das M.R., Hossain T., Mia M.B., Ahmed J., Kariman A.S., Hossain M.M. (2013). Fruit setting behavior of passion fruit. Am. J. Plant Sci..

[7] NDA-ARC (1999). Growing Granadillas.

[8] Rao B.N., Jha A.K., Deo C., Kumar S., Roy S.S., Ngachan S.V. (2013). Effect of irrigation and mulching on growth, yield and quality of passion fruit (*Passiflora edulis* Sims.). J. Crop Weed.

[9] Fischer I.H., Lourenco S.A., Martins M.C., Kimati H., Amorim L. (2005). Seleção de plantas resistentes e de fungicidas para o controle da podridão do colo do maracujazeiro causada por *Nectria hematococca*. Fitopatol. Bras..

[10] McKnight T. (1951). A wilt disease of the passion vines (*Passiflora edulis*) caused by a species of *Fusarium*. Queensl. J. Agric. Sci..

[11] Garcia E., Paiva D., Costa J., Portugal A., Ares A. (2019). First report of Fusarium wilt caused by *Fusarium oxysporum* f. sp. passiflorae on Passion Fruit in Portugal. Plant Dis..

[12] Liberato J.R., Costa H. (2001). Doenças fúngicas, bacterianas e fitonematóides. Maracujá: Tecnologia de produção, Pós-Colheita, Agroindústria, Mercado.

[13] Rooney-Latham S., Blomquist C.L., Scheck H.J. (2011). First report of Fusarium wilt caused by *Fusarium oxysporum* f. sp. passiflorae on Passion fruit in north America. Plant Dis..

[14] Fischer I.H., Rezende J.A.M. (2008). Diseases of passion flower (*Passiflora* spp.). Pest Technol..

[15] Li D.F., Yang J.Q., Zhang X.Y., Sun L.F. (1993). Identification of the pathogen causing collar rot of passion fruit in Fujian. Acta Phytopathol. Sin..

[16] Ploetz R.C. (1991). Sudden wilt of passionfruit in southern Florida caused by *Nectria haematococca*. Plant Dis..

[17] Viana F.M.P., Costa A.F., Freire F.C.O., Cardoso J.E., Viana F.M.P. (2003). Doenças do maracujazeiro. Doenças de Fruteiras Tropicais de Interesse Agroindustrial.

[18] Bezerra J.L., De Oliveira M.L. (1984). Damping-off of passion fruit caused by *Rhizoctonia* sp. [*Passiflora edulis*]. Fitopatol. Brasil..

[19] Young B.R. (1970). Root rot of passionfruit vine (*Passiflora edulis* Sims.) in the Auckland area, New Zealand. J Agric. Res..

[20] Lombard L., Sandoval-Denis M., Lamprecht S.C., Crous P.W. (2019). Epitypification of *Fusarium oxysporum* clearing the taxonomic chaos. Persoonia.

[21] Crous P., Lombard L., Sandoval-Denis M., Seifert K., Schroers H.-J., Chaverri P., Gené J., Guarro J., Hirooka Y., Bensch K. (2021). *Fusarium*: More than a node or a foot-shaped basal cell. Stud. Mycol..

[22] Zhao X., Li H., Zhou L., Chen F., Chen F. (2020). Wilt of *Acer negundo* L. caused by *Fusarium nirenbergiae* in China. J. For. Res..

[23] Bennett R.S., Davis R.M. (2013). Method for rapid production of *Fusarium oxysporum* f. sp. vasinfectum chlamydospores. J. Cotton Sci..

[24] De Carvalho J.A., de Jesus J.G., Araujo K.L., Serafim M.E., Gilio T.A.S., Neves L.G. (2021). Passion fruit (*Passiflora* spp.) species as sources of resistance to soil phytopathogens *Fusarium solani* and *Fusarium oxysporum* f. sp. passiflorae complex. Rev. Bras. Frutic..

[25] Silva A.S., Oliveira E.J., Haddad F., Jesus O.N., Oliveira S.A.S., Costa M.A.P. (2013). Variação genética em isolados de *Fusarium oxysporum* f. sp. passiflorae com marcadores AFLP. Sci. Agric..

[26] Nirenberg H.I. (1981). A simplified method for identifying *Fusarium* spp. occurring on wheat. Can. J. Bot..

[27] White T.J., Bruns T., Lee S., Taylor J.L., Innes M.A., Gelfand D.H., Sninsky J.J., White T.J. (1990). Amplification and direct sequencing of fungal ribosomal RNA genes for phylogenetics. PCR Protocols: A Guide to Methods and Applications.

[28] Carbone I., Kohn L.M. (1999). A method for designing primer sets for speciation studies in filamentous ascomycetes. Mycologia.

[29] Liu Y.J., Whelen S., Hall B.D. (1999). Phylogenetic relationships among ascomycetes: Evidence from an RNA polymerse II subunit. Mol. Biol. Evol..

[30] Kumar S., Stecher G., Li M., Knyaz C., Tamura K. (2018). MEGA X: Molecular evolutionary genetics analysis across computing platforms. Mol. Biol. Evol..

[31] Swofford D.L. (2003). PAUP*: Phylogenetic Analysis Using Parsimony (*and Other Methods), v. 4.0b10.

[32] Hillis D.M., Bull J.J. (1993). An empirical test of bootstrapping as a method for assessing confidence in phylogenetic analysis. Syst. Biol..

